# Apport de l'endoscopie digestive dans l'hypertension portale de l'enfant: à propos de 68 cas

**Published:** 2012-06-28

**Authors:** Mounia Lakhdar Idrissi, Abdeladim Babakhoya, Moustapha Hida

**Affiliations:** 1 Unité de gastro-entérologie, Service de pédiatrie, Département mère-enfant, CHU Hassan II, Fès, Maroc

**Keywords:** Endoscopie, enfant, hypertension portale, ligature, varice

## Abstract

**Introduction:**

L'hypertension portale n'est pas exceptionnelle chez l'enfant. L'hémorragie digestive en est une complication redoutable pouvant mettre en jeu le pronostic vital. Cette hémorragie, pouvant être isolée, confie à l'examen endoscopique un intérêt diagnostique majeur. L'endoscopie digestive haute a également un intérêt pronostique et thérapeutique incontournable. L'objectif de ce travail était d'analyser les aspects endoscopiques de l'hypertension portale, faire une corrélation entre ces aspects et le risque hémorragique éventuel et mettre en évidence le rôle de l'endoscopie dans le traitement et la surveillance.

**Méthodes:**

Notre étude est une analyse rétrospective de 135 endoscopies digestives hautes effectuées chez 68 enfants atteints d'hypertension portale sur une période de 8 ans.

**Résultats:**

L'endoscopie a permis de mettre en évidence les varices œsogastriques dans 55 cas (80.9%). Elle était le premier moyen diagnostique de l'hypertension portale chez 5 patients ayant présenté une hémorragie digestive isolée. Elle a permet aussi d'apprécier le risque hémorragique qui est étroitement lié au stade des varices œsophagiennes et à la présence des varices tubérositaires. Neuf enfants ont bénéficié de la ligature élastique des varices œsophagiennes avec un taux de succès de 89%.

**Conclusion:**

L'oesogastroscopie recherchant et traitant les varices œsogastriques est indispensable dans les hypertensions portales de l'enfant. Inversement, nous soulignons son intérêt majeur en matière diagnostique de l'hémorragie digestive isolée de l'enfant ou la découverte de varices pose à posteriori le diagnostic de l'hypertension portale.

## Introduction

L'hypertension portale (HTP) est définie par une élévation de la pression dans le système porte au dessus de 15 mmHg, ou par une élévation du gradient de pression porto-cave au-delà de 5mmHg. Celle-ci est liée à l'augmentation de la résistance à l’écoulement du sang dans le système porte, le plus souvent d'origine pré-hépatique (cavernome porte) et intra hépatique (cirrhose). Son évolution clinique est dominée par les complications essentiellement hémorragiques liées à la rupture des varices œsophagiennes et/ou tubérositaires. L'endoscopie est l'examen le plus performant pour mettre en évidence ces varices, identifier les patients à haut risque hémorragique, et assurer une meilleure prise en charge thérapeutique.

## Méthodes

Notre étude est une analyse rétrospective de 135 endoscopies digestives hautes effectuées chez 68 enfants atteints d'HTP au service de pédiatrie du CHU Hassan II de Fès durant une période de 8 ans (2003 à 2010). Les données cliniques et endoscopiques sont recueillies à partir des dossiers des malades et registres des comptes rendus de l′endoscopie digestive haute.

L’étude a inclue tous les cas, qu'ils aient saigné ou pas, ayant une HTP déjà confirmée par la clinique et/ou les données échographiques et les cas d'HTP diagnostiquée après un examen endoscopique réalisé pour une hémorragie digestive.

Le but de ce travail est d'analyser les aspects endoscopiques de l′HTP, faire une corrélation entre ces aspects et le risque hémorragique éventuel et mettre en évidence le rôle de l′endoscopie dans le traitement de l'HTP et la surveillance.

La ligature élastique des varices œsogastrique était le seul moyen thérapeutique endoscopique pratiqué dans cette série. Les vidéo endoscopes utilisés sont à vision axiale avec un béquillage quadridirectionnel permettant d′explorer en totalité le tractus digestif supérieur. On a utilisé l'endoscope pédiatrique dans le but diagnostic et celui de type adulte pour la ligature; les endoscopes qui ont un canal opérateur de 2,8 mm de diamètre étant les seuls qui permettent la réalisation d′actes d′endoscopie thérapeutique.

Pendant le déroulement de l'acte interventionnel, tous nos malades étaient mis sous anesthésie générale avec ou sans intubation trachéale. Les produits utilisés étaient le propofol, le midazolam et les gazs allogènes comme le sévoflurane et l'halothane. Après le geste, les patients restaient pendant une durée minimale de 5 heures dans la salle de réveil sous surveillance étroite de la fréquence respiratoire, du pouls et de la saturation en oxygène.

## Résultats

L′âge de nos patients était compris entre 2 mois et 16,5 ans. La moyenne d′âge était de 8,9 ans. Le sexe ratio était presque égal avec 35 garçons et 33 filles. La notion de consanguinité était révélée dans 12 cas (17,6%): 3 cas de maladie de Wilson, 2 cas de cavernome porte, 1 cas de syndrome d'Alagille, 1 cas de cirrhose et 5 cas d’étiologie inconnue.

La splénomégalie était présente chez 69% des patients. C’était le signe clinique le plus fréquemment retrouvé. L'atteinte hépatique était cliniquement évidente chez 08 patients (11,8%). L′hémorragie digestive était présente chez 21 malades (29.5%) et représentée essentiellement par l′hématémèse (16 cas). Le tableau complet d′ HTP n′était présent que chez 11 patients dont 7 avaient des varices œsophagiennes (VO) à l′endoscopie; quatre d'entre eux avaient présenté une hémorragie digestive haute.

Les étiologies de l′HTP étaient déterminées dans 42 cas (62%) et dominées par les blocs intra hépatiques dans 31cas (cirrhoses). Les blocs sous hépatiques ont pris la deuxième place avec 10 cas (cavernomes portes). Le bloc sus hépatique a été noté chez un seul malade (un syndrome de Budd-Chiari). L′hémorragie digestive était plus fréquente dans le groupe cavernome porte que dans le groupe du bloc intra hépatique (70% contre 22.5% respectivement).

**Figure 1:**
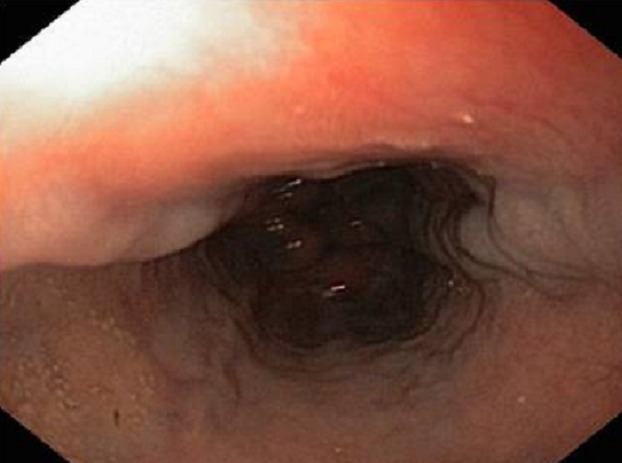
Les varices œsophagiennes stade I (photo du service de pédiatrie, CHU Hassan II, Fès)

**Figure 2:**
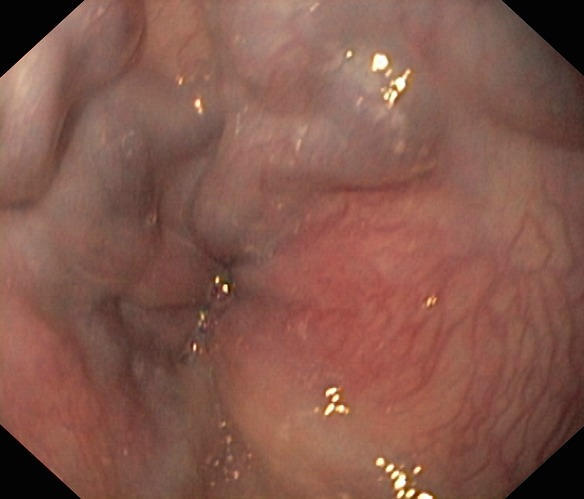
Les varices œsophagiennes stade II (photo du service de pédiatrie, CHU Hassan II, Fès)

**Figure 3:**
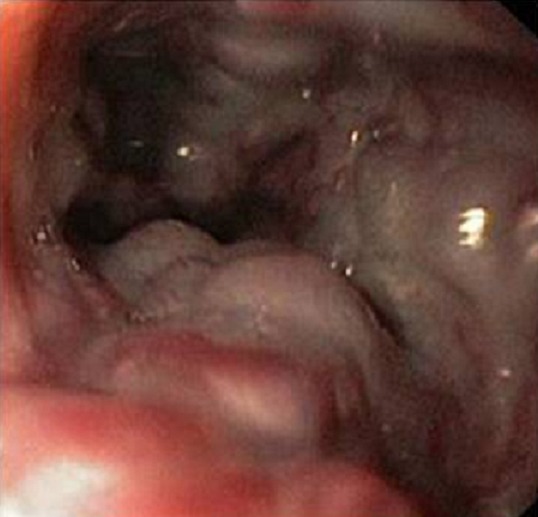
Les varices œsophagiennes stade III avec des signes de la série rouge (photo du service de pédiatrie, CHU Hassan II, Fès)

**Figure 4:**
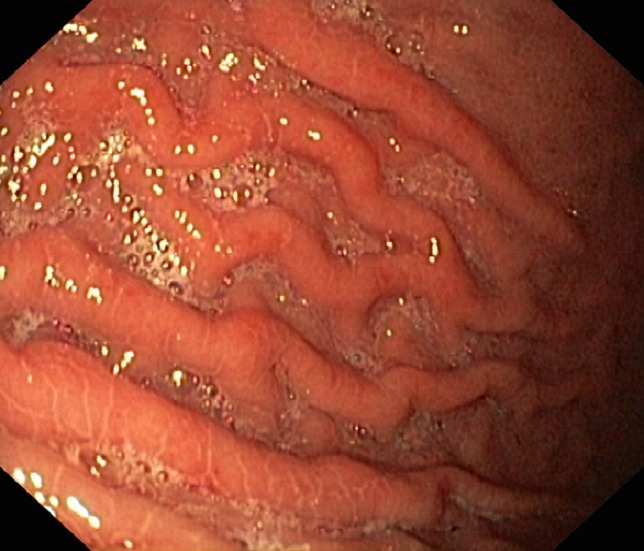
La gastropathie hypertensive (photo du service de pédiatrie, CHU Hassan II, Fès)

La fibroscopie a permis de mettre en évidence les varices œsogastriques dans 80,9 % des cas. Les VO étaient de stade I (VO1) dans 22 cas (Figure 1), stade II (VO2) dans 23 cas (Figure 2) et stade III (VO3) dans 10 cas (Figure 3). Les varices tubérositaires (VT) ayant été notées dans 9 cas étaient toujours associées à des VO (Tableau 1). L'examen endoscopique a permis aussi de préciser, sur les VO, les signes de la série rouge qui étaient la présence de tâches rouges cerise dans 9 cas, des tâches purpuriques et des zébrures dans 05 cas. La gastropathie hypertensive était retrouvée dans 23,5 % des cas (Figure 4). La découverte des VO avait posé à posteriori le diagnostic de l'HTP chez 5 patients ayant présenté une hématémèse isolée. Plus d′un tiers des patients ayant des varices oesogastriques ont saigné, sans pour autant avoir la preuve de l′intrication de ces varices dans le saignement. L′étude du risque hémorragique de nos patients était faite en tenant compte de la taille des varices, de l′aspect de la muqueuse oesophagienne, de la présence d′une lésion gastrique associée, et de la présence de VT. Le risque hémorragique était, en effet, associé à des VO stade III dans 41,4% des cas, à une muqueuse œsogastrique anormale dans 14,5% des cas, et surtout à la présence des VT dans 44,5% des cas (Tableau 2).

**Tableau 1:** Aspects endoscopiques observés dans notre série

	VO			Aspect normal	VT	Gastropathie hypertensive
	VO1	VO2	VO3			
Nombre de cas	23	19	13	10	9	16
%	33,8	27,9	19,1	14,7	13,2	23,5

VO : varices œsophagiennes ; VT : varices tubérositaires

**Tableau 2:** Lésions et stades de varices en cas d'hémorragie digestive

Lésions	Nombre de cas	% sur le nombre de malades ayant saigné
**VO 3**	04	20
VO 3 + VT	01	05
VO 3+ gastropathie hypertensive	01	05
VO 3+ VT+ gastropathie hypertensive	02	05
VO 3 + gastrite nodulaire	01	10
VO 3+ lésions gastriques purpuriques	01	05
**VO 2**	01	05
VO 2 + muqueuse œsophagienne congestive	01	05
VO 2 + gastrite erythémateuse diffuse	01	05
VO 1 + gastrite nodulaire	05	25
VO 1+ VT + gastrite nodulaire	01	05
VO 1 + ulcère gastrique	01	05
**Total**	**20**	**100**

VO : Varice œsophagiennes

**Figure 5:**
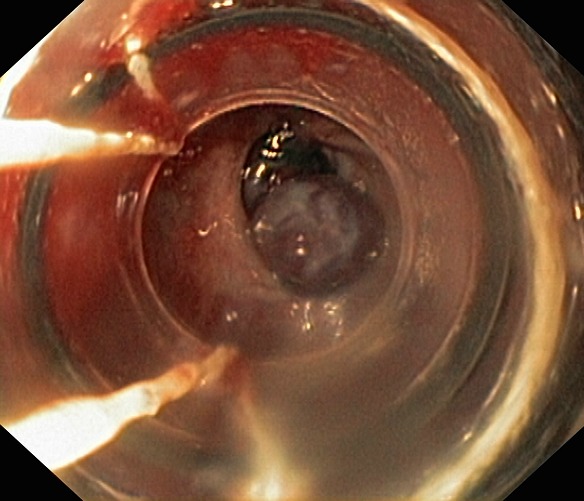
La ligature d'une varice œsophagienne (photo du service de pédiatrie, CHU Hassan II, Fès)

La ligature élastique des varices cardio-tubérositaires a constitué le seul mode thérapeutique endoscopique dans notre série. Neuf enfants ont bénéficié de cette intervention. Il s'agit de 6 cas de cavernome porte, un cas d'hépatite auto-immune et 2 cas d'HTP d’étiologie indéterminée. Au total, 21 séances ont été réalisées soit une moyenne de 3 séances par malade. Pour 8 d'entre eux, la ligature avait concerné des VO stade III et dans l'autre cas, elle a été faite pour des VT (Tableau 3). La ligature était faite en urgence initialement dans 3 cas ayant présenté des hémorragies digestives de grande abondance et ce après stabilisation de l’état hémodynamique. Pour les 6 autres, elle était toujours programmée (Figure 5).

**Tableau 3:** Les indications de la ligature des varices œsogastriques

Varices œsogastriques	Nombre	Pourcentage
**VO 3**	8	89
**VO 3+ VT**	3	33.5
**VO 2+ VT**	1	11

VO: varices œsophagiennes

A court terme, six patients traités par la ligature élastique avaient bien évolué avec arrêt de saignement immédiat pour les 3 cas oú la ligature était faite en urgence. Une récidive hémorragique précoce (2 jours après la 1ère ligature) a été notée chez un malade qui a nécessité aussitôt une deuxième séance. Un malade de 4 ans, cirrhotique, a présenté à deux reprises des pics fébriles ayant duré moins de 48 heures et avec un bilan infectieux négatif. Un cas de douleurs rétro sternales transitoires a été noté. L’évolution à long terme était favorable chez 7 enfants (pas de récidive hémorragique, régression des varices œsogastrique). La récidive hémorragique était signalée dans un seul cas après un intervalle de 5 mois. On a déploré un décès chez une fille de 12 ans ayant une hépatite auto-immune. Ce décès, survenu loin du geste endoscopique, était suite à une encéphalopathie hépatique et une décompensation ascitique.

Tous les malades de notre série ont bénéficié d'un traitement ß bloquant (Propranolol) avec une bonne tolérance après un suivi régulier. Les autres thérapeutiques: vitamine K1, transfusion de plasma frais congelé et diurétiques étaient prescrites en fonction de la symptomatologie clinique.

## Discussion

L′endoscopie digestive est une technique désormais utilisée en routine en gastroentérologie pédiatrique. Elle représente un moyen diagnostique sûr, à la double condition qu′elle soit réalisée par un endoscopiste habitué à l′enfant et que le matériel soit adapté. L′endoscopie digestive permet également des actes thérapeutiques qui tendent à se développer mais qui doivent rester réservés aux opérateurs expérimentés.

Le diagnostic endoscopique des lésions de l′hypertension portale digestive repose sur des critères précis. Les VO, apparaissent comme de longs cordons veineux de couleur généralement bleutée, situés juste au-dessus du cardia. Elles sont classées en trois stades: le stade I lorsque les VO s′aplatissent à l′insufflation, le stade II pour les VO ne s′effaçant pas lors de l′insufflation et qui sont non jointives et le stade III pour les VO jointives, obstruant la lumière œsophagienne [1–3]. Bien qu′ils ne soient pas prédictifs du risque de rupture des varices, le nombre de VO, leur siège sur la circonférence oesophagienne, ainsi que leur hauteur mesurée en centimètre depuis la ligne en « Z » (jonction des muqueuses oesophagienne malpighienne blanchâtre et gastrique cylindrique rose) sont à décrire. Les signes de la série rouge applicables à tout type de varices, sont de description variée: macules rouges cerise, zébrures, voussures érythémateuses, zone ecchymotique, télangiectasies diffuses ou encore des lésions purpuriques [4, 5].

La description des VT bénéficie d′une classification simplifiée: grade I pour une présence probable, grade II en cas de certitude du fait, de leur différenciation nette des gros plis gastriques, et de la présence de signes rouges [6].

La gastropathie vasculaire de l′HTP est secondaire au syndrome hyperkinétique avec hypervolémie et vasodilatation. Elle se présente en deux degrés: modéré devant un aspect en maille ou mosaïque, sévère devant un piqueté purpurique, des ectasies vasculaires antrales et des érosions [7–9].

Affirmer la rupture des VO est possible dans prés de la moitié des cas, que l′œsophagoscopie révèle un saignement en «jet» ou en « nappe » des varices, ou plus souvent un caillot plaquettaire blanc adhérant à une varice, ou une érosion ecchymotique [10, 11]. Les 3 ou 4 derniers centimètres de l′œsophage sont le site électif du saignement par rupture de VO. En l′absence de tels aspects, certains signes endoscopiques ont fait la preuve de leur valeur prédictive positive de rupture: 1) VO très tendues, 2) Aspect congestif, ecchymotique ou télangiectasique de la muqueuse œsophagienne, 3) Varices gastriques [12]. La lésion hémorragique est identifiée avec certitude dans 70 à 80% des cas lorsque la fibroscopie est réalisée dans les délais courts soit moins de 24 heures en post hémorragique [13, 14].

L′endoscopie permet aussi de contrôler l′efficacité du traitement médical par le propranolol [14, 17]. Nos malades bénéficient toujours de contrôles endoscopiques réguliers; d'autres séances de ligature seront éventuellement programmées en fonction des lésions observées. L’évaluation de l'efficacité du traitement chirurgical fait aussi appel à l'exploration endoscopique. Ainsi, les signes d'HTP sont soit inchangés, dans ce cas le shunt porto-systémique est probablement thrombosé ou peu fonctionnel, soit la fibroscopie montre un aplatissement des varices et/ou la disparition de la congestion de la muqueuse oesophagienne, dans ce cas le shunt est probablement perméable mais insuffisamment fonctionnel, soit la disparition complète des VO, de la congestion muqueuse et des varices gastriques, dans ce cas, on peut affirmer que le shunt est perméable et fonctionnel [15, 16]. Dans notre série, aucun malade n’était opéré.

Sur le plan thérapeutique, la sclérose endoscopique des varices œsophagiennes est la méthode la plus ancienne et qui a fait la preuve de son efficacité chez l′adulte. Chez l'enfant, la sclérothérapie des VO est efficace dans la prévention des récidives des hémorragies mais elle est associée à des complications significatives [18–21]. La ligature élastique des varices est de pratique récente chez l′enfant et tend à se substituer à la sclérothérapie [11, 22–24].

Chez l′enfant une étude récente contrôlée et randomisée comparant la ligature à la sclérose a montré que la ligature avait de meilleurs résultats concernant la vitesse d′éradication des VO (4 séances contre 6), la diminution des récidives hémorragiques (4% contre 25%), et des complications (4% contre 25%) [25]. Plusieurs études ont montré que la ligature endoscopique des varices en association avec une prophylaxie à long terme avec le propranolol est une technique efficace et sure pour éradiquer l′hémorragie digestive par rupture des VO chez l′enfant [14, 17, 26]. Le taux de complications est faible; des ulcérations superficielles, des douleurs rétro-sternales ou une dysphagie transitoire sont possibles, mais les sténoses oesophagiennes sont rares et aucune perforation œsophagienne n′a été rapportée. L′association sclérose et ligature ne semble pas plus efficace que la ligature seule [27]. Cependant, l'une des limites de la ligature tient au diamètre du ligateur mis sur l′embout distal de l′endoscope qui rend délicate la manœuvre d′introduction du fibroscope à la bouche œsophagienne de l'enfant.

L′hémostase par oblitération des VO par injection intra-variqueuse de colles acryliques (N-butyl-2-cyanoacrylate) est rarement pratiquée, sauf lorsque l′hémorragie digestive est consécutive à la rupture de varices fundiques. Les varices gastriques chez l′enfant sont peu mentionnées dans les études, bien que leur incidence soit de 12 à 27% [13]. Pour les rares autres localisations (varices rectales ou duodénales), les méthodes endoscopiques sont réalisées en fonction de l′appréciation de l′opérateur: sclérose ou ligature élastique.

## Conclusion

L'endoscopie digestive haute a un intérêt majeur dans l'hypertension portale chez l'enfant comme chez l'adulte. Elle constitue le premier moyen diagnostique dans les cas inaugurés par l'hémorragie digestive. Le traitement des varices oesophagiennes fait appel aux techniques d'endoscopie interventionnelle quelles soient la sclérose ou la ligature élastique des varices. La ligature élastique est devenue la technique de choix pour une éradication plus rapide des varices et pour la prévention des récidives hémorragiques.
